# Pyorrhœa Alveolaris

**Published:** 1895-10

**Authors:** J. E. Cravens

**Affiliations:** Indianapolis, Ind.


### International Dental Journal.

Vol. XVI. October, 1895. No. 10.

### Original Communications.[1]

### PYORRHŒA ALVEOLARIS.[2]

BY DR. J. E. CRAVENS, INDIANAPOLIS, IND.

In the progress of the lesion or disease known as pyorrhœa alveolaris (Riggs's disease), the only tissue really destroyed is the alveolar process and principally that participating in the septæ; and, save the exhibition of some incidental calculary deposit, there is nothing in the appearance of freshly extracted roots, from affected sockets, that affords the slightest suggestion of the existence or action of the disease in consideration; assuredly, then, the conclusion forces itself upon one that pyorrhœa alveolaris is not, strictly, a dental disease,—*perhaps not a dental disease at all*,—a possible assumption that may be strengthened by the fact that the pericementum is not a participant, but is affected to some extent by some of the results of the disease.

The tract of pericementum involved in a pus "pocket" is not discovered in congestion, whatever may be the condition of the remainder of the membrane in those parts of the socket intact.

Pyorrhœa alveolaris is of itself little more than a condition, a

[1] The editor and publishers are not responsible for the views of authors of papers published in this department, nor for any claim to novelty, or otherwise, that may be made by them. No papers will be received for this department that have appeared in any other journal published in the country.

[2] Read before the Massachusetts Dental Society at the annual meeting, June 5 and 6, 1895.

result, however, of a periosteitis that finds origin on the surface of the alveolar ridge, and later by extension invades the alveolus; therefore, to study the pathology and seek out the etiology and history of this ostensible disease, one must consider the affections of the periosteum.

PERIOSTEITIS.

The causes of periosteitis usually are not so obscure as to prove impossible of discovery, but there are cases whereof the cause can not be assigned. A surprising parallel to the principal facts of pyorrhœa alveolaris is presented in an extended essay on osteitis, periosteitis, etc., by R. H. M. Dawbarn, in the "Reference Hand-Book of the Medical Sciences," pp. 381 to 385, inclusive.

In the following quotation from his language on the subject, Mr. Dawbarn appears to recognize little clinical difference between periosteitis and osteitis,—viz.:

"It is of little clinical value to classify inflammations of bone into osteitis, osteomyelitis, and periosteitis, from an anatomical stand-point, since primary periosteitis, with exception of the traumatic and syphilitic varieties, is rarely observed."

In the quotations to follow, from the same authority, it is hoped to show to the satisfaction of my auditors, the close application of the pathology and progress of periosteitis to the symptoms and facts of the hitherto mysterious (?) pyorrhœa alveolaris. The double character of periosteitis should be held in mind by students of pyorrhœa alveolaris, as this characteristic plays a conspicuous part in the tragedy of cure. The statement of Dawbarn is,—

"Chronic, non-infective periosteitis may be either fibrous or ossifying. In the former [fibrous] there is much increase in the amount of connective-tissue, and the thickened membrane adheres unusually closely to the bone; in the latter [ossifying] we have as a result an ossific deposit."

Recurring briefly to a preliminary statement of mine, to the effect that pyorrhœa alveolaris is a condition resulting from periosteitis, and that alveolar process is the only structure really destroyed in the progress of the disease,—we may profitably invade the alveolus in pursuit of the parallel where we find a cushion or mass of soft and highly vascular tissue lining the greater limit of the "pocket." This mass is the apparatus of destruction or repair as the case may be. The existence of such a mass of soft tissue is a clinical fact familiar to practitioners who have essayed treatment of Riggs's disease. Destruction of alveolar process and consequent enlargement (pocketing) upon one or more aspects of the root of a tooth, will continue as long as the mass of granulation tissue endures undisturbed; that this is a fact also of chronic periosteitis is verified by Dawbarn as follows:

"In periosteitis and rarefying osteitis, the absorption of bone is thought by some pathologists to be caused by the presence of certain large multinucleated cells, the myeloplaxes of Robin, called, from this idea, osteoclasts. . . . Other pathologists disbelieve that these large cells possess any such power, and attribute the absorption to the influence of the new granulation tissue always present in these cases and lying in contact with the bone."

Proceeding upon the hypothesis that the real lesion or disease in a given case of pyorrhœa alveolaris is an affection of the periosteum, and that the usual facts and manner of destruction of bone practically are the same in the alveolus as elsewhere, except there be modifications due to comparative density of the tissues acted upon, we may seek the etiology of the disease upon the same hypothesis. The primary manifestation of inflammation in these cases occurs in the periosteum on the ridge and about the orifice of the socket; for descriptive convenience I have called this primary condition *orbicular periosteitis.* Should orbicular periosteitis continue on the surface of the bone, there will be a persistent case of *gingivitis*, which may prove to be serious or may not; but, should the periosteitis penetrate the socket, as it usually will in time if unchecked, then the condition known as Riggs's disease, or phagedenic pericementitis, or pyorrhœa alveolaris, or gouty inflammation of the peridental membrane, or whatever name you may wish it to be known by, will obtain.

### ETIOLOGY.

In reference to the origin of inflammation of bone and of the periosteum, Dawbarn gives a variety of causes, nearly all of which are possible in the mouth. As to osteitis Dawbarn says,—

"Inflammation of a bone may be induced by simple traumatism, . . . by extension from a periosteitis, by extension from arthritis, by exposure to cold, or to action of certain poisons, as phosphorus, mercury, syphilis; by pressure, by eruptive fevers, typhoid possibly acting as a primary and certainly as a predisposing cause."

Also,—

"Periosteitis may originate from traumatism, either simple or compound, and in character may be simple [that is aseptic] or septic from the presence of micro organisms."

In the mouth there are not wanting abundant causes for orbicular periosteitis: pressure of a persistent nature, even of so mild a character as that due to salivary calculus being sufficient, if under the margin of the gum; injury by dam-clamps may be either pressure or traumatism, or even both; impinging ligatures, badly adjusted regulating apparatus, ill-fitting crowns and wabbling plates, or traumatic injuries immediately about the sockets, due to any of a hundred and one simple carelessnesses, such as splinters of wooden tooth-picks thrust into the gum tissue; woundings by pins or needles that are used for picking the teeth or carried carelessly in the mouth, a most pernicious habit with many women; and often, far too often, careless and awkward manipulation of dental instruments that probably are far from clean. It is possible that what we recognize as aggravated gingivitis is due to "simple" or "aseptic" periosteitis, while that which is "septic" is responsible for invasion of the sockets and destruction of bone, the formation of the pus "pockets," and other facts of pyorrhœa alveolaris; in either case the disease is local and "non-infective."

### PROGRESS OF DISEASE.

As to the manner of destruction of the bone forming the alveoli, the parallel between pyorrhœa alveolaris, osteitis, and periosteitis may again be applied, in the following from Dawbarn:

"It is thought that the granulations [new granulation tissue] evolve an acid. Formerly it was believed that lactic acid was the solvent. Tillmann's later researches seem to show that it is the carbonic acid in the blood which dissolves the bone."

The fact that in all cases of pyorrhœa alveolaris there is notable inconstancy of gingival membrane, not only of that about sockets that are pocketing, but prevailing generally about all teeth in the mouth, leads to an inference that such inconstancy is prerequisite to establishment of the disease in consideration. The inconstancy may be due to any of many things and conditions; the subject may be anæmic, run down by overwork, by dissipation, or as a condition following protracted fever, or illness of any character, all tending to encourage local lesion or disease.

### PROGNOSIS.

If inconstancy of gum margins were general and of long standing in a particular case, it would indicate constitutional obliquity extending far back of any observed local conditions. Such obliquity may be either hereditary or acquired; an acquired obliquity usually is temporary, being amenable to treatment, besides nature is self-asserting in such cases; that which is hereditary offers less encouragement, because seldom reducible by treatment, and nature cannot be depended upon as an ally. Therefore, if there were evidence that a patient had inherited a constitutional obliquity that was manifested in the mouth by this inconstancy of gingival tissue, I should regard the subject as probably difficult to cure of pyorrhœa alveolaris, but not incurable; and that under conditions arising from personal negligence of sanitary care of the teeth, generally by the patient, there might be recurrence of the disease after every evidence of cure. I do not believe that pyorrhœa alveolaris ever is inherited, or that it ever is incurable.

Practitioners generally assent to the opinion that this disease is oftener noted between majority and middle life; however, the infrequency of the disease in old age is accounted for by the frequent loss of affected teeth after middle age, and cannot be justly attributed to a supposed superiority of tissue structure of our progenitors. Within a year I have had under treatment three subjects, two girls and one boy, who were each about seventeen years old; two had Riggs's disease over three years before coming to me, and the other had developed the disease within a year; all badly affected, and difficult to cure because of recurring gingivitis.

Dr. A. W. Harlan, of Chicago, told me of a case in his practice where the patient was but nine years old, and the symptoms of pyorrhœa alveolaris were well defined; so, it appears that neither old age nor youth enjoys exemption from this destroyer of teeth. Observation has convinced me that young subjects are bad subjects; their gum tissue tends to ready recurrence to gingivitis in discouraging frequency, which is the exception in mature years; add to this their frequent lack of appreciation of affairs in their mouths, an indisposition to persist in the treatment even when rendered comparatively painless; and that other fact, that parents so often prove to be poor allies to the dentist. But young subjects may be cured for all that.

Pyorrhœa alveolaris is very erratic; it is not always general in the mouth, but frequently is found about several sockets, isolated from each other by several intervening teeth, and not confined to the same jaw. Within a week I have examined a case of a lady, where the whole right side of each jaw was affected, not one tooth having escaped from the median line to the wisdom teeth. There is no sign of the disease upon the left side of that mouth, and exactly the same condition was observed in that case three years ago. I have a man patient over fifty years of age, who came to me in August, 1893, with a bad case of this disease about the second and third inferior molars; there was extensive outward displacement of the second molar, due to the disease, which brought the crown into false and painful articulation; within two weeks after the surgical operation the crown resumed its proper position, and remains so to-day, doing good service as a masticator. There have been no subsequent care of that case, no return of the disease, and no sign of any other teeth being affected; I saw this case in April last.

Now, as to constitutional treatment of pyorrhœa alveolaris: two physicians' wives were under my care for this disease two or three years since; their husbands essayed to help by constitutional measures, but without appreciable effect. Other cases have come under my observation, and I am convinced that constitutional measures are beneficial to the local treatment of pyorrhœa alveolaris, to the same extent that they may be to treatment of ingrown toe-nails, no less, and certainly no more.

### SYMPTOMS.

The symptoms of pyorrhœa alveolaris are divisible into *constant* and *inconstant.*

### CONSTANT SYMPTOMS.

Pus always is present in untreated "pockets," but cannot always be demonstrated at once, because of protecting bone, which prevents pressure of a finger effectively.

The mucous membrane overlying a "pocket" is glazed, the gum hard and thickened; the color being modified from red to a violet, particularly that of the festoons.

The *health line* dips towards the effected sockets; just in proportion as health is restored in the parts.

In cases of long standing and numerous "pockets" there is report of feverish discomfort of gums at almost all hours; disagreeable taste emanating about the affected teeth, particularly on awakening in the morning, frequent lassitude, impaired digestion.

These symptoms are not always constant.

### INCONSTANT SYMPTOMS.

Frequent lancinating pains in affected sockets; sometimes regional neuralgia.

Slow recession of gum on labial and lingual aspects; a secondary growth of violet tinted tissue from bases of shrunken festoons, between the teeth, rather indicating an early stage of the disease.

Distressing night pains, when abed, sometimes described by the sufferer as a *drawing sensation* in the "pockets;" relief is obtained only by sitting up; this symptom indicates profound and longstanding "pockets."

The teeth become elongated, loose, pushed out of the arch or rotated in position with soreness; the last item indicating congestion of pericementum or periosteum, or both, where there is still attachment that has not been disturbed by the pocketing.

Sallow skin, hollow orbits, languor, indigestion, livid lips, ropy saliva, breath smells of pus.

One must not expect to find all these symptoms at once. I have discovered extensive establishment of pyorrhœa alveolaris where nearly all the usual symptoms were wanting.

### DIAGNOSIS.

The diagnosis, of course, is based upon the known symptoms. The observations of the patient should first be ascertained, and the extent to which they comport with stated symptoms should be noted; such information may guide the dentist where usual indications are obscure.

Observe closely for gingival inflammation and sponginess of gum and characteristic violet modification from red, particularly of festoons.

Apply firm pressure with a finger or other means upon the gum at cervix and bases of festoons, and watch for possible expression of pus.

Test all gingival borders with a thin, flat probe, for in many cases the "pockets" are concealed.

Interrogate as to gastric disturbance due to ingested pus, weakness, lassitude, facial neuralgia, bad taste, etc.

### TREATMENT.

The object of treatment should be to cure. The first step to that end is to thoroughly cleanse the mouth. Remove all visible (salivary) calculus and polish off ordinary stains. All cavities of decay should be put in good sanitary condition and temporarily stopped with gutta-percha, to remain so until after the treatment shall be completed.

Having located a "pocket" and determined its extent and form, select a suitable instrument and proceed to remove whatever of calculus may be attached to the root within the "pocket." But the operation should invariably be preceded by certain preparatory measures, such as thoroughly douching the "pocket" with water of 140° F., which should be projected into the opening with considerable force. The forcible current and the high temperature of the water will effectually displace all pus as well as any loose *débris* from the "pocket," and the pounding effect of the hot fluid will stimulate receptivity of soft tissues, enlarge capillaries, and permit relief of congestion; this use of hot water immediately causes a whitening of the congested gum, and the patient is apt to report a sense of relief; the promotion of receptivity is advantageously demonstrated by the rapid and almost complete obtundation by cocaine, without hypodermic injection; it is seldom that a patient will submit to a second surgical sitting unless there has been local anæsthesia, and the dentist cannot be excused for failure to at least try to mitigate pain. In most cases the operation may be rendered almost if not quite painless.

Remember that there is not the slightest danger of overdoing the hot-water treatment as a preliminary, an intermediate, and even a final measure.

The choice and care of syringes is an important factor in treating pyorrhœa alveolaris; most "pockets" are impenetrable by the nozzles that come with the ordinary dental syringes, and often it is necessary that the point of the nozzle shall reach the ultimate depth in order to discharge cocaine at the place of greatest sensibility.

Remember that water scalds at 150° F., that 140° F. is as effective as 150°, and absolutely safe.

After or during removal of the *pyonal calculus* from a root, I lacerate the mass of "new granulation tissue" freely, even bringing away patches and shreds of it, together with portions of loosened and folded pericementum that I may discover at or near the apex; the object of this laceration, etc., is to force a change from chronic and destructive form of periosteitis, to the acute and bone-making character that is essential to a cure of the disease and obliteration of the "pockets." Permit me here to revert to language of Dawbarn, already quoted, as follows:

"In periosteitis and rarefying osteitis, the absorption of bone is thought by some pathologists to be caused by the presence of certain large multinucleated cells, the myeloplaxes of Robin, called, from this idea, osteoclasts. . . . Other pathologists disbelieve that these large cells possess any such power, and attribute the absorption to the new granulation tissue always present in these cases, and lying in contact with the bone."

Before passing from the surgical act to another topic in relation, let me assure my auditors that obliteration of a "pocket" does not always follow a successful operation, nor is such a result essential to a cure; but a partial filling of the excavation is.

### LOCAL OBTUNDATION.

Owing to the present limited knowledge of local obtundents, cocaine promises better for this purpose than anything else, but requires extended application to produce desired results; experience has led me to adopt two solutions, one in water, the other in chloroform. Both solutions should be saturated; a poor article of chloroform will dissolve more of the alkaloid than the best, and I am not sure but it makes the more effective preparation. A saturated solution of cocaine in pure water amounts to about two hundred per cent., while a saturation in best chloroform attains eight per cent. with difficulty. The water solution probably is a dangerous preparation to handle in the mouth, and much caution should be practised with it; my method is to not use more than a single drop at a time, if possible, and take precaution that it does not escape about the mouth. This cannot be prevented always, but to guard against harmful effects there may always be at hand a supply of brandy or whiskey with which to lave the mucous membrane of the mouth; the local relief from cocaine is usually prompt and complete from such means, but the stimulant must be held in the mouth until the benumbed parts begin to feel a sense of burning from the alcohol. I presume that a diluted preparation of alcohol of any other flavor would answer this purpose; in fact, I often employ a druggist's bay-rum that is merely a highly flavored preparation of alcohol of fifty-five per cent. strength. The advantage of brandy or whiskey over other forms of alcohol is that it may be swallowed after laving the mouth sufficiently, thus forestalling unfavorable effects upon the brain, which patients are liable to experience after leaving the office of the dentist.

After the surgical procedure, follow immediately with a solution of sulphuric acid, made of one part commercial acid to ten parts of water, the common battery solution. Guard the parts to prevent ingress of saliva until there has occurred a blackening of clotted blood about the orifice of the "pocket;" then dry the clots with cotton, and protect with a sort of antiseptic varnish called stérésol,[1] which must be flowed over the surface of clot and adjacent membrane and evaporated for a minute or two. This stérésol is a tenacious dressing for operations in the mouth, and will hold safely for several days if undisturbed by brush or pick. Stérésol was mentioned to me by Dr. I. B. Davenport, of Paris, who said that it was used by Dr. Michaels of that city to protect the "pockets" after operations for pyorrhœa alveolaris. Its formula is accessible to pharmacists, and the preparation is compounded for me in Indianapolis for my personal use, and I have found it very effective in preserving antiseptic condition of cases after operations in pus "pockets."

SECONDARY TREATMENT.

After about five days following a surgical sitting, a patient should return for a secondary treatment, which should consist in first washing the parts with water, heated as before to 140° F.; second, increasing the temperature to near 150° F. and continue washing; if any portion of a "pocket" remains open, it should be douched out as at first with a forcible current; but the operator should be careful not to disturb any granular tissue with the end of the nozzle of the syringe I have found that there is much benefit to be obtained from following this secondary douching with a ten-per-cent. solution of nitrate of silver. This completes the secondary treatment.

As the dilute sulphuric acid sometimes is quite painful, if the effect of cocaine has passed off, and the stains of nitrate of silver are very objectionable and annoying, I have tried to dispense with these preparations, but always to my regret. As to these, I am still hopeful of some satisfactory substitutes, but they belong to the future of this treatment. I should gladly congratulate some dentist of Massachusetts on such a discovery.

Immediately upon a case of this disease coming under my care, I require a discontinuance of alkaline substances in the shape of

---

[1] STÉRÉSOL.—An adhesive, antiseptic varnish, for use in the mouth.

℞ Shellac, ℥ix;

Benzoin, ʒiiss;

Balsam tolu, ʒiiss;

Carbolic acid-crystal, ℥iij, ʒiij;

Oil cinnamon, ʒiss;

Saccharin, ʒiss;

Alcohol, q. s. ad Oij.

Digest several days and decant or strain. (Stable prep.)

dentifrices, soap, or medicines, because they encourage precipitation of the salts of common tartar in the mouth, and thus place the treatment at disadvantage. I recommend frequent rinsing of the mouth with diluted bromo-chloralum, which is very cleansing and a powerful astringent; it is a valuable auxiliary; also, I advise the use of pulverized sulphur as a dentifrice, which is wonderfully effective for polishing the teeth, but usually its flavor and dryness render it not very seductive. The sulphur may be discontinued after completion of treatment, but the bromo-chloralum should be continued for several months but in greater dilution; it is very sour.

I always expect a patient to recover promptly and without failure, unless my surgical operation has been hastily and imperfectly performed.

When pus is no longer formed and the disease is obliterated, a case is cured. The dentist cannot make new bone; *a cure is all that reasonably may be expected of the dentist.* Creative economy alone provides for reproduction of tissue.

In conclusion, permit me to say that experience leads me to believe that pyorrhœa alveolaris is non-infective; it is not constitutional, although a tendency to it may be; it is not always symmetrical; it may be associated with gout, but is no part of it; it is often associated with affections of the Schneiderian membrane, and may complicate them, but exists in full vigor after such affections have been reduced; it could not well escape association with nasal catarrh in a country where that has come to be regarded as a test of our nationality.

Finally, pyorrhœa alveolaris is curable, and there is as little tendency to its recurrence as of any other local lesion after cure. The mask of mystery so long worn by this disease has been torn away. I congratulate humanity and my brother dentists.

